# Bioinformatic analysis of the potential common pathogenic mechanisms for gastric precancerous lesions and *Helicobacter pylori*

**DOI:** 10.3389/fgstr.2025.1598916

**Published:** 2025-10-07

**Authors:** Liufeng Yi, Tiantong Jiang, Siyu Tao, Nachuan Li, Yuan Ding, Meng Li, Shaoli Wang, Zhen Liu

**Affiliations:** ^1^ Department of Gastroenterology, Guang’anmen Hospital of Chinese Academy of Chinese Medical Sciences, Beijing, China; ^2^ Graduate School, Beijing University of Chinese Medicine, Beijing, China

**Keywords:** bioinformatics analysis, gastric precancerous lesions, *Helicobacter pylori*, hub genes, differentially expressed genes

## Abstract

**Objective:**

Gastric precancerous lesions (GPL) represent a critical stage in the progression from gastritis to gastric cancer (GC). Helicobacter pylori (H. pylori) infection is a significant etiological factor exacerbating the inflammatory-cancerous transformation. The host immune status is a central regulatory mechanism for GPL, while persistent H. pylori infection drives changes in the immune microenvironment (IME). However, the potential pathological link between GPL and H. pylori remains unclear. This study aims to identify common differentially expressed genes (DEGs) between GPL and H. pylori using bioinformatics analysis, thereby elucidating their shared pathogenic mechanisms.

**Methods:**

DEGs were extracted from GPL datasets (GSE87666) and H. pylori datasets (GSE60427) sourced from the Gene Expression Omnibus (GEO) database through GEO2R and R software. Protein-protein interaction (PPI) networks were generated using the STRING database and analyzed with Cytoscape software to identify hub genes. The diagnostic value of these hub genes was evaluated through Receiver Operating Characteristic (ROC) curves and Area Under the Curve (AUC) analysis, validated with datasets GSE130823, GSE60662, and GSE5081. Immune infiltration analysis of key genes was conducted using the CIBERSORT algorithm and ssGSEA. Gene mutation analysis was carried out using the cBioPortal database, and small molecule drugs were detected using the Connectivity Map (CMap) database.

**Results:**

The analysis revealed 189 DEGs. Functional enrichment analysis highlighted pathways related to immune system regulation, leukocyte migration and chemotaxis, cytokine-cytokine receptor interaction, and chemokine signaling. Seven hub genes were identified: IL6, FCGR3A, CCL3, CXCR4, CXCL9, CCL4, and CCR1. High AUC values for these hub genes indicated their potential for predicting disease occurrence.

**Conclusions:**

This research identified seven hub genes closely related to GPL and H. pylori, elucidating potential mechanisms of disease progression, particularly emphasizing the roles of the IME and chemokine activity. These findings may provide insights for identifying disease biomarkers, inhibiting the “inflammation-cancer” transformation, and preventing GC. Furthermore, the targets and molecular mechanisms identified in this study require further experimental validation to confirm their therapeutic potential.

## 1 Introduction

The latest cancer report from the International Agency for Research on Cancer indicates that GC ranks fifth globally in terms of both incidence and mortality (1), making the reduction of these rates a pressing public health challenge. GPL are recognized as a key stage in the carcinogenesis pathway of Correa intestinal-type GC (2). Reports suggest that GPL, as an early stage of GC, has an annual incidence of 0.6% in cases with mild to moderate atypical hyperplasia and 6% in cases with severe atypical hyperplasia (3). Reversing these lesions and containing the disease at the GPL stage is crucial for reducing early GC incidence.

H. pylori is a spiral-shaped bacterium that colonizes the gastric mucosa, causing chronic inflammation and ulceration (4). As a Class I carcinogen for GC, this bacterium infects over 50% of GC patients (5). H. pylori eradication therapy has been shown to prevent GPL progression, with virulence factors serving as a critical factor in altering the IME (6). However, the exact pathways through which H. pylori triggers inflammation and facilitates GC progression remain poorly understood (7). This study aims to identify DEGs between GPL and H. pylori and analyze related functional pathways to explore their relationship and provide new insights for early GC diagnosis and prevention.

## 2 Data and methods

### 2.1 Data collection and source

The datasets of GPL and H. pylori were obtained from the GEO (http://www.ncbi.nlm.nih.gov/geo/) database using the keywords “gastric precancerous lesions” and “Helicobacter pylori,” respectively. The datasets GSE87666 and GSE60427 were selected based on the criteria of species ‘Homo sapiens’ and a sample size greater than 10. Dataset GSE87666 included data from 16 patients with GPL and 6 patients with early gastric cancer (EGC), while GSE60427 included data from 8 H. pylori-negative and 24 H. pylori-positive patients. Additionally, the GPL dataset GSE130823 and the H. pylori datasets GSE60662 and GSE5081 were selected to validate the hub genes.

The GSE87666 and GSE60427 gene chip datasets were downloaded using the GEOquery package in R (8) and subsequently normalized with the limma package to ensure comparability (9). Following annotation, the DEGs were visualized using volcano plots. The results from the two datasets were intersected using the ggplot2 and VennDiagram packages to identify DEGs between GPL and H. pylori tissues, with the results presented in a Venn diagram. Additionally, significantly expressed molecules were visualized using heatmaps (10, 11). **Table 1** provides specific details of the datasets.

**Table 1:** Sample information for the datasets.

GEO name	Sample size	Control group	Patient group	Platforms	Published
Gastric precancerous lesions					
GSE87666	22	16	6	GPL17077	Aug 21, 2017
GSE130823	47	31	16	GPL17077	Mar 15, 2020
Helicobacter pylori					
GSE60427	32	8	24	GPL17077	Jun 01, 2015
GSE60662	12	4	8	GPL13497	Aug 23, 2014
GSE5081	32	16	16	GPL570	Jun 04, 2008

### 2.2 Functional enrichment analysis

The DEGs with converted IDs were subjected to enrichment analysis using the clusterProfiler package in R, including biological process (BP), cellular component (CC), and molecular function (MF). Based on a significance threshold of P < 0.05, the 10 most significant terms were identified and visualized as bar plots for Gene Ontology (GO) enrichment and chord diagrams for Kyoto Encyclopedia of Genes and Genomes (KEGG) enrichment.

### 2.3 Construction of PPI network

The PPI network was generated from the STRING database with a medium confidence score set at 0.400 and disconnected nodes hidden, and then imported into Cytoscape (Version 3.9.1). The MNC and EPC algorithms were applied to obtain the top 10 scored genes, and the results were combined to determine the core genes of the relevant pathways.

### 2.4 Construction and analysis of ROC curves

The expression levels of 10 hub genes were evaluated for their diagnostic value using ROC analysis with the pROC package in R, visualized with ggplot2, and validated with datasets GSE130823, GSE60662, and GSE5081. The AUC value typically ranges from 0.5 to 1. A higher AUC value indicates superior diagnostic performance. In this study, hub genes with an AUC > 0.65 were selected as having high diagnostic value.

### 2.5 Immune cell infiltration analysis

The abundance of 22 common immune-infiltrating cells types in the samples was estimated using the CIBERSORT algorithm (12). Variation in immune cell infiltration between GPL and GC patient samples, as well as between H. pylori-negative and -positive samples, were assessed using box plots. Additionally, after downloading and organizing the stomach adenocarcinoma (STAD) data from The Cancer Genome Atlas (TCGA, https://portal.gdc.cancer.gov), the ssGSEA algorithm and Spearman analysis were applied to evaluate the relationship of hub genes with immune infiltration levels in TCGA-STAD, using markers for 24 immune cell types from the literature, with results visualized as heatmaps using ggplot2 (P < 0.05) (13, 14).

### 2.6 Mutations of hub genes in GC patients

The cBioPortal (http://www.cbioportal.org) database facilitates the exploration, analysis, and visualization of cancer genomics and clinical data. Data from 625 GC patients were utilized to analyze mutation profiles of the identified hub genes.

### 2.7 Identification of potential therapeutic small molecules by CMap database

The CMap database is designed to elucidate the relationships between gene expression and drug action, identifying potential treatments and clarifying drug mechanisms. Disease co-DEGs are imported into the CMap database to conduct correlation analyses between drug expression profiles and disease expression profiles. The entries classified as trt_cp underwent screening, and the 15 compounds demonstrating the most significant negative correlation were recognized as potential therapeutic agents based on the norm_cs correlation score.

## 3 Results

### 3.1 Identification of DEGs

**Figure 1:**
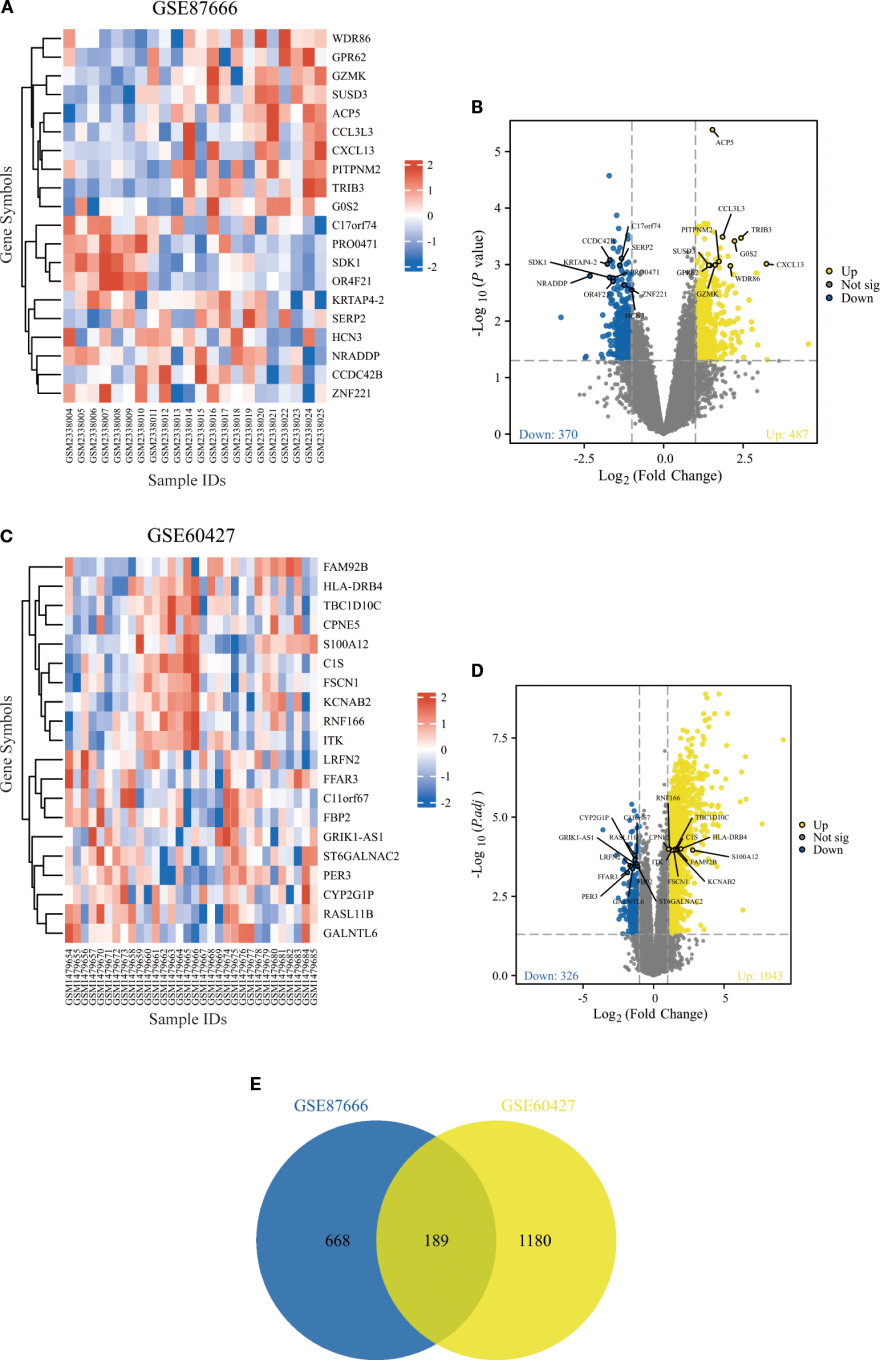
Identification and screening of DEGs. **(A, B)** Heatmap **(A)** and volcano plot **(B)** representing DEGs from dataset GSE87666. **(C, D)** Heatmap **(C)** and volcano plot **(D)** representing DEGs from dataset GSE60427. **(E)** Venn diagram of common DEGs between GSE87666 and GSE60427.

From the GPL dataset GSE87666, 857 DEGs were identified. The heatmap illustrates the expression of the top 20 up- and down-regulated genes across samples (**Figure 1A**), while the volcano plot delineates 487 up-regulated and 370 down-regulated genes (**Figure 1B**). From the H. pylori dataset GSE60427, 1369 DEGs were obtained, comprising 1043 up-regulated and 326 down-regulated genes (**Figures 1C, D**). Ultimately, 189 common DEGs were identified through the intersection of the GPL and H. pylori datasets (**Figure 1E**).

### 3.2 Gene enrichment analysis

**Figure 2:**
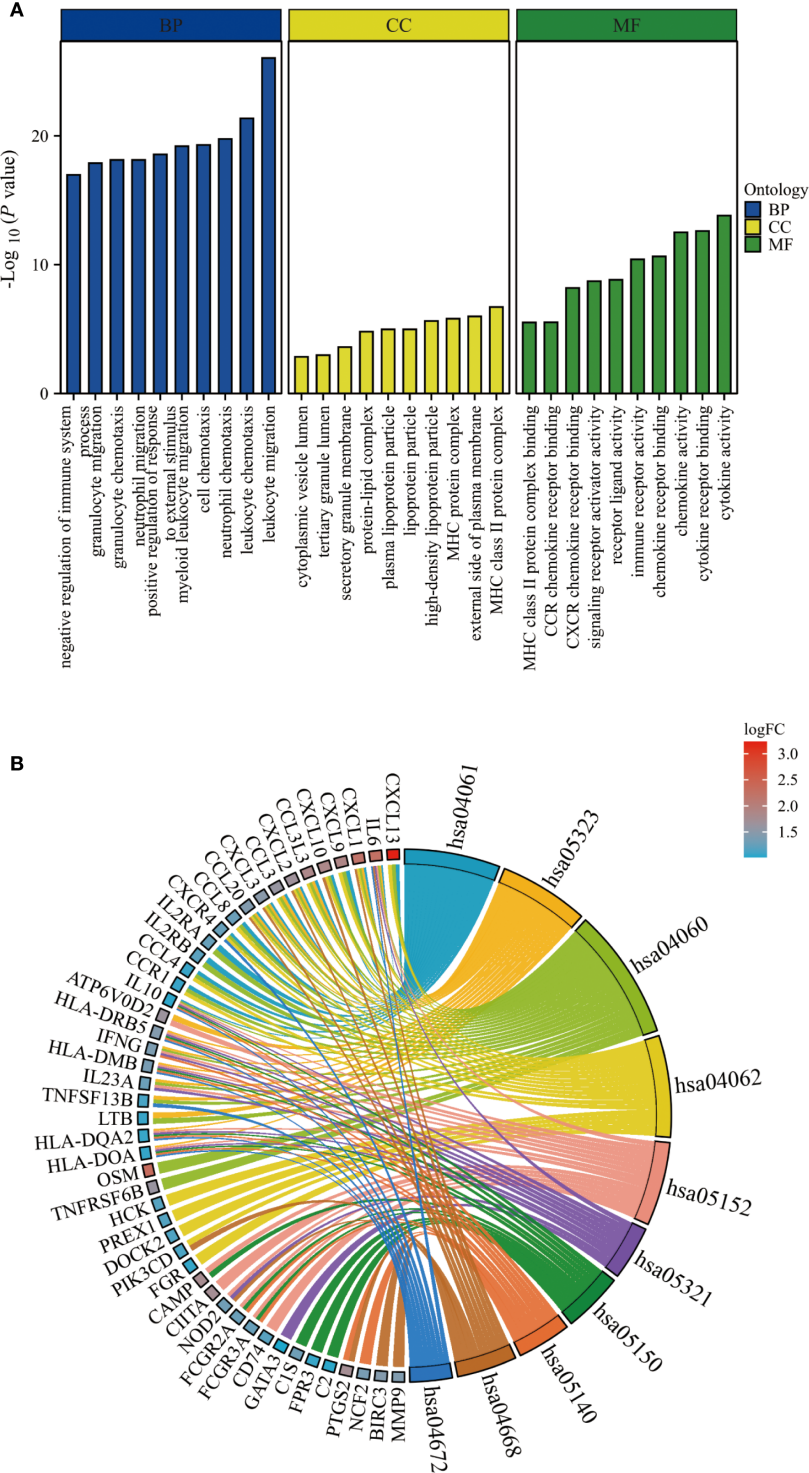
Functional enrichment analysis. **(A)** The top 10 items of GO enrichment analysis. **(B)** The top 10 items of KEGG enrichment analysis.

With a significance threshold of P < 0.05, GO enrichment analysis indicated that the BP of DEGs were linked to leukocyte migration and chemotaxis, positive regulation of response to external stimulus, and negative regulation of immune system processes. Regarding CC, DEGs were primarily expressed in the MHC protein complex, lipoprotein particles, and tertiary granule lumen. MF was predominantly enriched in chemokine receptor binding, immune receptor activity, and MHC class II protein complex binding (**Figure 2A**). KEGG enrichment analysis highlighted pathways including chemokine signaling, cytokine-cytokine receptor interaction, and TNF signaling, further confirming their key roles in immune responses (**Figure 2B**).

### 3.3 PPI network construction and hub gene identification

**Figure 3:**
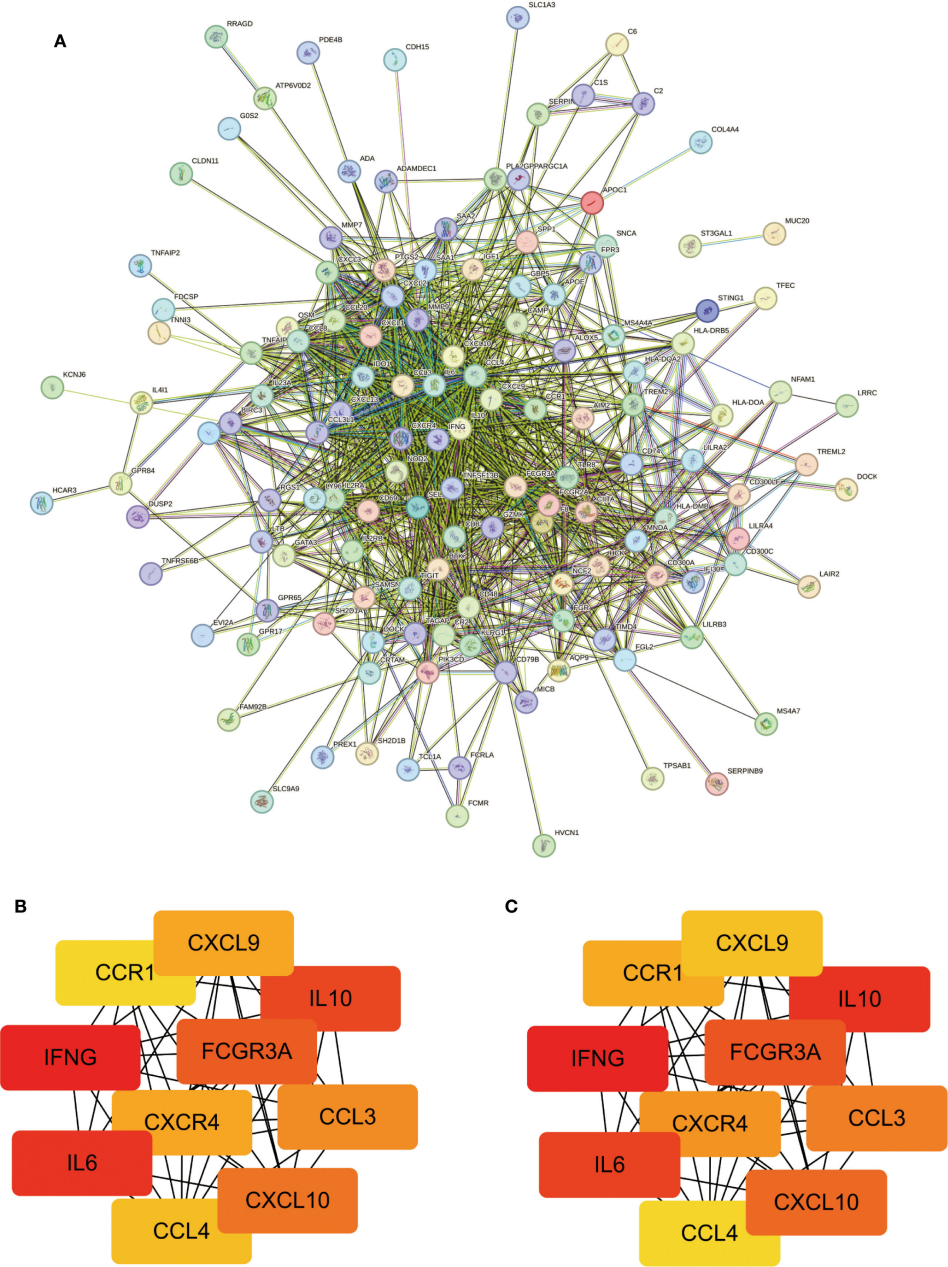
Significant modules and functions of PPI network. **(A)** Construction of the network using the STRING database. **(B, C)** Identification of ten genes using the MNC/EPC algorithms.

A PPI network comprising 169 node genes and 1084 edges was constructed based on the STRING database (**Figure 3A**). The MNC and EPC algorithms of the CytoHubba were employed to select the top 10 scoring genes (**Figure 3B**). This analysis identified 10 hub genes: IFNG, IL6, IL10, FCGR3A, CXCL10, CCL3, CXCR4, CXCL9, CCL4, and CCR1.

### 3.4 ROC curve analysis of hub genes

**Figure 4:**
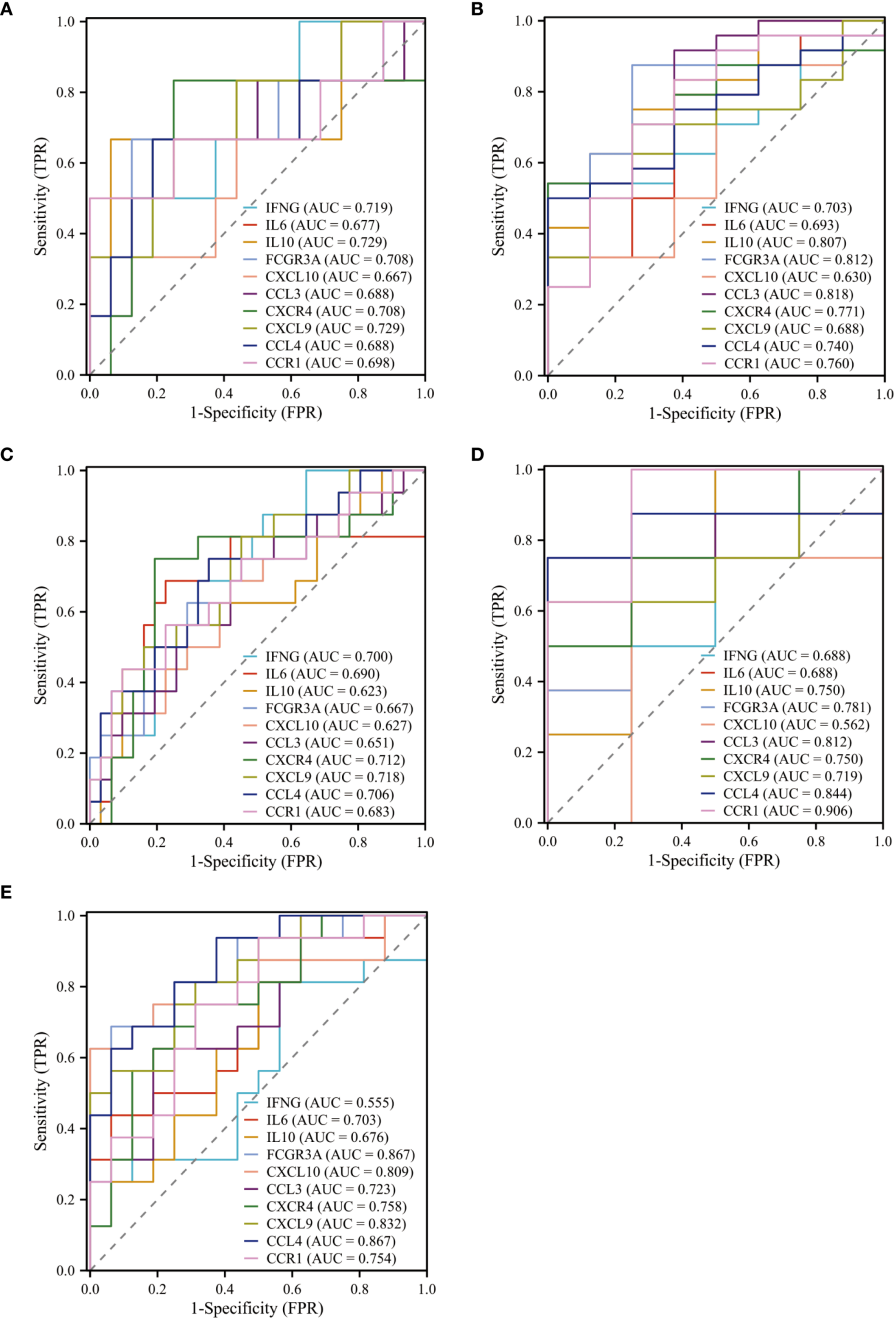
ROC curves evaluate the diagnostic validity of ten genes in five datasets. **(A, B)** ROC curve and AUC value of ten genes in the training sets GSE87666, GSE60427. **(C–E)** ROC curve and AUC value of ten genes in the validation sets GSE130823, GSE60662, GSE5081.

The diagnostic predictive value of genes was evaluated using ROC curves across different datasets. In the GPL GSE87666 dataset, the AUC values for IFNG (0.719), IL6 (0.677), IL10 (0.729), FCGR3A (0.708), CXCL10 (0.667), CCL3 (0.688), CXCR4 (0.708), CXCL9 (0.729), CCL4 (0.688), and CCR1 (0.698) all exceeded 0.65 (**Figure 4A**). Similarly, in the H. pylori GSE60427 dataset, the AUC values for IFNG (0.703), IL6 (0.693), IL10 (0.807), FCGR3A (0.812), CXCL10 (0.630), CCL3 (0.818), CXCR4 (0.771), CXCL9 (0.688), CCL4 (0.740), and CCR1 (0.760) were evaluated, with all genes except CXCL10 showing AUC values above 0.65 (**Figure 4B**).

The AUC values in the three validation sets equally demonstrated satisfactory predictive performance. In the GPL GSE130823 validation set, IL10 (0.623) and CXCL10 (0.627) had AUC values below 0.65 (**Figure 4C**). In the H. pylori GSE60662 validation set, CXCL10 (0.562) had an AUC value below 0.65 (**Figure 4D**). In the H. pylori GSE5081 dataset, IFNG (0.555) had an AUC value below 0.65 (**Figure 4E**). The remaining seven core genes—IL6, FCGR3A, CCL3, CXCR4, CXCL9, CCL4, and CCR1—met the screening criterion of AUC > 0.65 in all datasets, suggesting their potential as diagnostic markers or therapeutic targets for GPL associated with H. pylori.

### 3.5 Immune cell infiltration analysis

**Figure 5:**
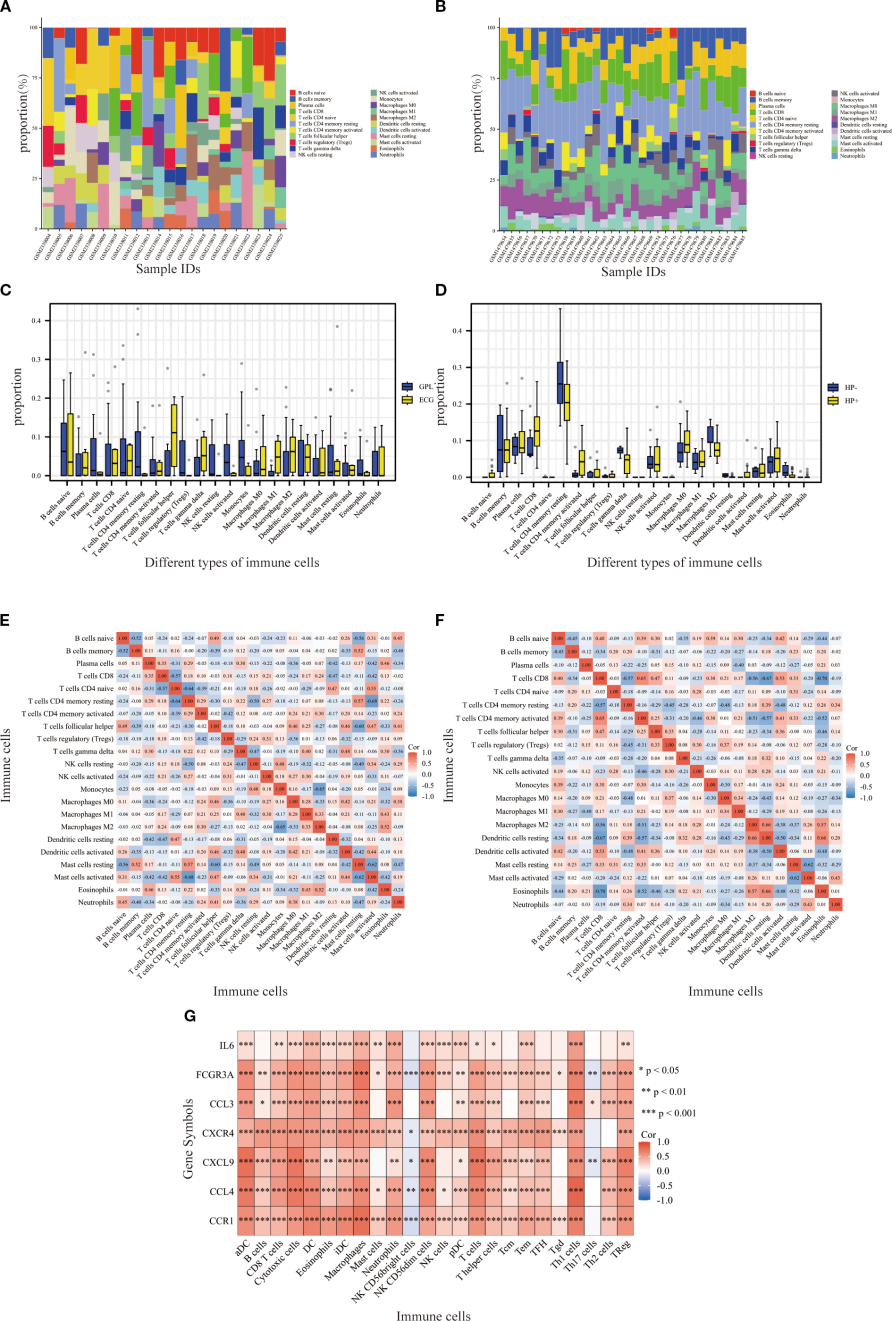
Immunocyte infiltration analysis. **(A, B)** Stacked bar graphs showing the relative proportions of various immune cell types in the GPL and EGC groups, H. pylori-negative and H. pylori-positive groups. **(C, D)** Boxplots showing the infiltration of various immune cells in GPL GSE87666 and **(H)** pylori GSE60427 datasets. Blue represents GPL/H. pylori-negative patients, and yellow represents EGC/H. pylori-positive patients. **(E, F)** Heatmaps showing the correlation between the different types of immune cells across samples in the GSE87666 and GSE60427 datasets. **(G)** Heatmap showing the correlation between hub genes and immune cells according to the ssGSEA algorithm. *p<0.05, **p<0.01, ***p<0.001.

Significant differences in the IME were observed between the GPL and EGC groups. The abundance of plasma cells, CD4 memory resting T cells, regulatory T cells, resting NK cells, and activated NK cells was markedly decreased in the EGC group as opposed to the GPL group. In contrast, follicular helper T cells and M1 macrophages demonstrated higher expression in the EGC group (**Figure 5A**). Additionally, in the H. pylori-positive group, the activity of CD4 memory T cells was markedly elevated relative to the H. pylori-negative group (**Figure 5B**). Boxplots illustrated the infiltration levels of 22 immune cells across the samples (**Figures 5C, D**), with the correlation between resting NK cells and H. pylori status being zero in all samples. (**Figures 5E, F**). Based on the ssGSEA algorithm, the associations between hub genes and immune infiltration matrix data from TCGA-STAD was visualized in a heatmap. The hub genes were positively correlated with 22 immune cells, except for NK CD56 bright cells and Th17 cells (**Figure 5G**), indicating a strong infiltration relationship with TCGA-STAD immune cells.

### 3.6 Mutation analysis of the hub genes

**Figure 6:**
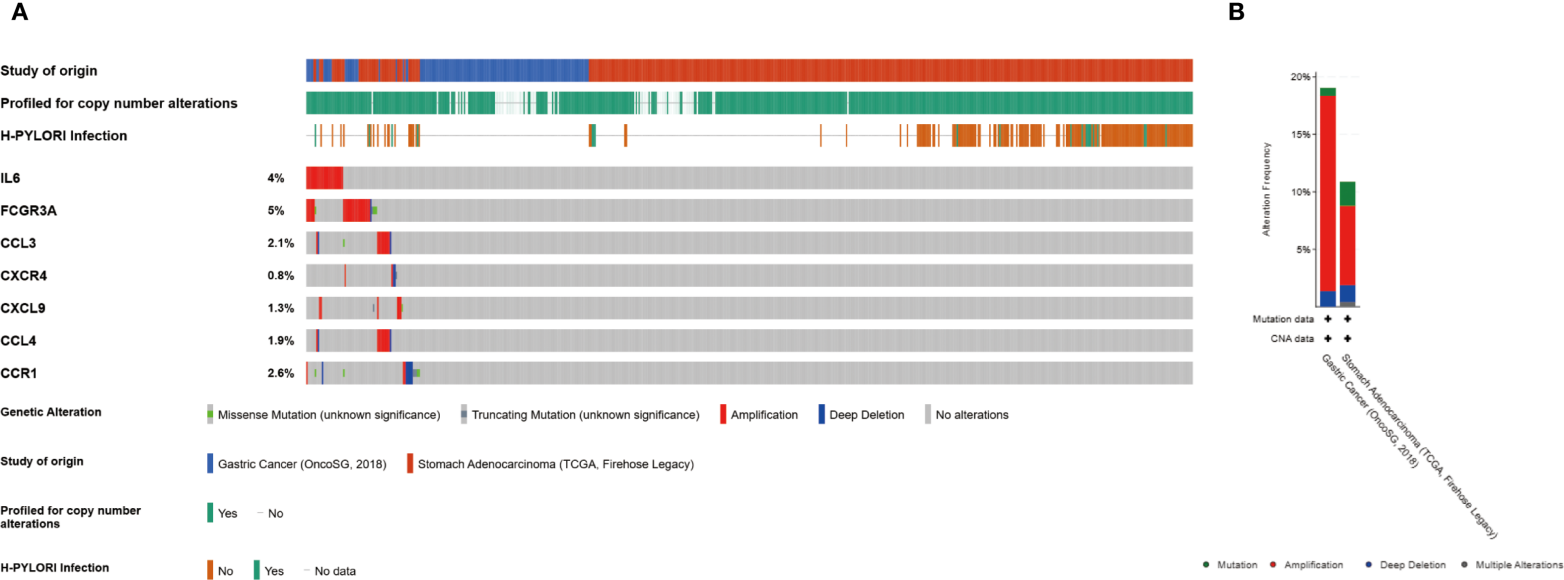
Genetic alterations of hub genes. **(A)** Representation of genetic mutations in seven genes. **(B)** The total frequency of alterations in seven genes.

The frequency of genetic alterations in seven genes was assessed through the cBioPortal (https://pmc.ncbi.nlm.nih.gov/articles/PMC10034022/) database. Among 625 GC cases, the mutation rates for IL6, FCGR3A, CCL3, CXCR4, CXCL9, CCL4, and CCR1 were identified as 4%, 5%, 2%, <1%, 1%, 2%, and 3%, respectively (**Figures 6A, B**). Amplification was the predominant alteration type. Additionally, the prevalence of H. pylori in GC cases in this study was 30.1%.

### 3.7 Screening of small molecule compounds targeting hub genes

Based on the CMap database, the 15 small molecule compounds with the highest inverse correlation with the common DEGs of GPL and H. pylori were identified, suggesting that these compounds may exhibit potential therapeutic efficacy (**Table 2**).

**Table 2:** Identification of candidate small molecule compounds using the CMap database.

ID	Name	MOA (description)	Norm_cs
BRD-K10671814	sulfaphenazole	Dihydropteroate synthase inhibitor	-2.1025
BRD-K13514097	everolimus	MTOR inhibitor	-2.0705
BRD-K73368362	BIX-02189	MEK inhibitor	-2.0341
BRD-K76617868	fasudil	Rho associated kinase inhibitor	-2.0077
BRD-K49685476	TTNPB	Retinoid receptor agonist	-1.996
BRD-K24656285	farnesol	FXR agonist|Monoamine oxidase inhibitor|NFKB activator|PPAR receptor agonist	-1.9841
BRD-A02180903	betamethasone	Glucocorticoid receptor agonist	-1.9748
BRD-K26521938	dinoprostone	Prostanoid receptor agonist	-1.9709
BRD-A69815203	cyclosporin-a	Calcineurin inhibitor	-1.969
BRD-K80778372	Ro-19-4605	GABA receptor agonist	-1.9662
BRD-A72483914	spiroxatrine	Serotonin receptor antagonist	-1.962
BRD-A45153512	bimatoprost	Prostanoid receptor agonist	-1.9614
BRD-K49865102	PD-0325901	MEK inhibitor|MAP kinase inhibitor|Protein kinase inhibitor	-1.9453
BRD-A29520968	acifran	Cholesterol inhibitor	-1.9436
BRD-K83189926	UNC-1215	L3MBTL antagonist	-1.9422

## 4 Discussion

H. pylori infection is a pivotal factor in the etiology of GPL. This infection induces persistent inflammation, significantly altering the expression patterns of gastric mucosal cells and immune cells, including multiple chemokines and chemokine receptors (15). The IME plays a pivotal role in H. pylori pathogenesis. Chronic infection leads to an imbalance within the immune system, with the gastric mucosal tumor microenvironment (TME) infiltrated by inflammatory cells such as tumor-associated macrophages (TAMs), neutrophils, dendritic cells (DCs), myeloid-derived suppressor cells, and natural killer (NK) cells. They release pro-inflammatory cytokines, chemokines, and reactive oxygen/nitrogen species, contributing to tissue damage, genomic instability, and GPL progression. In GPL, immune cells can target and eliminate abnormal proliferating cells, thereby inhibiting lesion progression. However, H. pylori infection induces immune escape, allowing abnormal cells to evade immune surveillance and promoting lesion progression (7).

The hub genes identified in this study—IL6, FCGR3A, CCL3, CXCR4, CXCL9, CCL4, and CCR1—play crucial roles in the immune system. These molecules regulate the migration, activation, and function of immune cells through complex interactions, contributing to inflammatory responses, immune surveillance, and tumor progression.

IL6, a multifunctional cytokine involved in infection responses, is closely associated with immune diseases and cancer. Tumor-promoting mesenchymal stromal cells (GC-MSCs) within the GC niche secrete large amounts of IL6, promoting the polarization of M2 macrophages, key immune cells in the tumor stroma (16). IL6 enhances the proliferation, migration, and invasion of GC cells through activation of the JAK2/STAT3 signaling pathway and upregulation of epithelial–mesenchymal transition (EMT)-related genes (17). H. pylori infection is reported to enhance IL6-mediated autocrine and paracrine positive feedback loops among macrophages and gastric epithelial, potentially contributing to GC development (18).

The Fcγ receptor IIIa (CD16a), encoded by FCGR3A, is primarily expressed on NK cells and monocytes. FCGR3A is associated with immune cell infiltration in the TME, potentially influencing the killing capacity of NK cells and monocytes against tumor cells, consequently affecting the immune surveillance and control of GPL (19). Single-cell studies have elucidated the role of FCGR3A in macrophage heterogeneity within the tumor microenvironment, revealing that elevated expression of characteristic genes in FCGR3A-type macrophages is significantly correlated with decreased overall survival in GC (20). Although no direct studies link H. pylori infection to FCGR3A, the impact of this bacterium on GC IME suggests that it may indirectly affect FCGR3A function or expression, thereby influencing GPL immune surveillance.

CCL3, also known as macrophage inflammatory protein 1-α, is a key chemokine that binds to specific cell surface receptors (e.g., CCR1, CCR3, and CCR5). It induces chemotaxis and activation of immune cells, promotes inflammatory cell infiltration, and influences tumor cell proliferation and apoptosis. H. pylori infection stimulates macrophages to express and secrete CCL3, disrupting the tight junctions of gastric epithelial cells and impairing the gastric mucosal barrier function. These disruptions create conditions conducive to further H. pylori invasion and GPL development (21).

The chemokine receptor CXCR4 is frequently upregulated in GC cells by H. pylori infection, hypoxia, and inflammatory factors. H. pylori infection induces gastric mucosal cells to secrete CXCL12, the ligand for CXCR4, thereby activating the receptor (22). This binding activates multiple signaling pathways, including PI3K/AKT and MAPK, which promote GC cell proliferation and migration. CXCR4 signaling also upregulates pro-angiogenic factors such as VEGF, providing nutrients for tumor growth. The synergistic activation of these pathways directly contributes to the malignant phenotype of GC cells (23).

In the GC microenvironment, CXCL9 is produced and secreted in response to inflammatory factors, including IFN-γ. CXCL9 binds to CXCR3, a receptor commonly expressed on GC cells, thereby activating the JAK2-STAT3 signaling pathway. This activation leads to increased PD-L1 expression on tumor cells, which in turn suppresses the function of CD8+ T cells and facilitates immune escape by GC cells (24). It was shown that H. pylori infection leads to increase the secretion of CCL3 by macrophages, which in turn induces CXCL9 secretion via the JAK1-STAT1 pathway (21).

CCL4, a macrophage inflammatory protein-1β, can destabilize the TME and anti-tumor immune function by recruiting Tregs and TAMs to act on other resident cells in the TME, such as fibroblasts, endothelial cells, which can participate in carcinogenesis and promote tumor growth (25). To date, no experimental studies have directly elucidated the role of CCL4 in GC and GPL. However, its expression appears to be linked to inflammatory responses. The chronic inflammatory response induced by H. pylori infection can stimulate gastric mucosal cells and immune cells, including monocytes, DCs, and lymphocytes to promote the expression of CCL4.

CCR1 serves as a major receptor for chemokines such as CCL3 and CCL4, directing the migration of immune cells to sites of infection, trauma, or abnormal proliferation. It also recruits myeloid-derived suppressor cells and M2-type tumor-associated macrophages, forming an immunosuppressive microenvironment that promotes the progression of GPL (26). As such, it serves as a pivotal link between inflammation and cancer.

The IME is closely associated with GC development. Tumors evade immune surveillance through various mechanisms, including enhancing negative immunoregulatory mechanisms and influencing antigen presentation. Immune cell populations significantly modulate immune responses and thereby exert crucial influence on GC, consistent with the enrichment analysis results in this study (27). No direct studies have yet elucidated the relationship between H. pylori infection and the NK cells resting. The level of NK cell infiltration in GC is negatively correlated with tumor progression, suggesting that reduced NK cell infiltration may be associated with poor patient survival.

Studies have reported that the immune microenvironment is closely related to the development of gastric tumors. Tumors use a variety of mechanisms to evade immune surveillance. These include enhancing negative immunoregulatory mechanisms and influencing antigen presentation. Immune cell populations play a key role in gastric tumorigenesis by modulating immune responses, consistent with the results of the enrichment analysis in this study. The GPL group is characterized by a high infiltration of activated NK cells, which are key mediators of innate antitumor immunity. In contrast, the GC group shows a negative correlation between NK cell infiltration and tumor progression, along with increased infiltration of M2 macrophages and neutrophils. M2 macrophages, which promote angiogenesis, tissue remodeling, and immune suppression, are indicative of a tumor microenvironment that facilitates tumor growth and immune evasion. These findings support the notion that there is a transition from robust antitumor immunity in the precancerous stage to a predominantly immunosuppressive state in gastric cancer. The progression from GPL to GC is further characterized by a decline in antitumor immune responses and a concomitant enhancement of pro-tumor immunity, suggesting potential targets for immunotherapeutic intervention.

The hub genes identified in this study exhibit negative correlations with NK CD56 bright cells and Th17 cells. It is reported that the increased infiltration of CD56bright cells in tumor tissues is higher than that in normal tissues (28). Single-cell RNA sequencing analysis has characterized heterogeneous cell populations in GPL and GC, revealing that the infiltration level and functional performance of NK cells in GPL may be related to the initiation of GC (29). High infiltration of Th17 cells and Tregs in GC tissues may disrupt the Th17/Treg balance, thereby affecting GC development. H. pylori infection has been demonstrated to promote Th17 cell differentiation and alter the Th17/Treg balance, promoting immune escape (30).

Hub genes demonstrate potential application value in the field of tumor immunotherapy. Specifically, IL6, CCR1, and CXCR4 are implicated in the recruitment and activation of immune cells in the TME. FCGR3A and CXCL9 may regulate cell function and the anti-tumor immune response. CCL3 and CCL4 are linked to tumor immune escape and immunotherapy resistance. These genes may offer a novel perspective and research direction for GC immunotherapy and provide a theoretical basis for developing immunotherapy strategies.

Hub genes are frequently observed to undergo copy number amplified in GC. High amplification levels are typically associated with overexpression of these genes, which may confer growth and proliferation advantages to GC cells. Additionally, H. pylori infection is detected in some samples, which may be linked to genetic variation and the development of GC. Identifying amplified genes may contribute to identifying potential therapeutic targets for GC, particularly in case of H. pylori infection.

Currently, there is a lack of sufficient clinical evidence to support the therapeutic efficacy of the compounds identified in this study against GC and GPL. Among these compounds, Sulfaphenazole, a traditional sulfonamide antibiotic used primarily for bacterial infections such as urinary tract infections, has been shown to reduce reactive oxygen species production by inhibiting CYP2C9, thereby alleviating tissue oxidative damage (31). Everolimus, an mTOR inhibitor, has demonstrated antitumor activity by inhibiting GC cell proliferation in both *in vitro* and *in vivo* studies (32). Clinical trials indicate that everolimus monotherapy achieves a disease control rate of 56% and improves progression-free survival in patients with advanced GC. Additionally, everolimus can ameliorate the inflammatory response in gastric epithelial cells caused by H. pylori infection (33). Fasudil, a Rho kinase (ROCK) inhibitor, is clinically used for preventing and treating cerebral vascular spasm. It relaxes smooth muscle and improves blood circulation by inhibiting the Rho/ROCK signaling pathway and exhibits anti-inflammatory and anti-fibrosis effects (34). However, direct clinical studies investigating Fasudil for gastric diseases are currently unavailable. Future research may focus on elucidating the mechanisms linking these compounds with H. pylori and GPL, while exploring their therapeutic potential.

## 5 Conclusion

Differential analysis of gene expression data from GPL and H. pylori reveals common clusters of DEGs. These clusters are significantly correlated with immune cell infiltration and substantially influence the immune response process, suggesting that immunotherapy may be effective in treating GC. Potential small molecule compounds have been screened; however, their mechanisms of action on GPL and H. pylori were not analyzed in this study. This study is based exclusively on bioinformatics analysis without experimental validation at the cellular, animal, or clinical sample level. The research on immune infiltration is limited to preliminary exploration and similarly lacks experimental validation. Therefore, the conclusions drawn remain to be supported by subsequent experiments to enhance their reliability.

## Data Availability

The original contributions presented in the study are included in the article/supplementary material. Further inquiries can be directed to the corresponding author.
